# 

**Published:** 1900-04-07

**Authors:** 


					a, iifli. ?? ice standard o! Highest purity;*'?Lancetv <" April Tan, 1900.
SpWaDBURY'S #?? M S??! CADBURY'S
No. .70i6. Vol. XXVIII. [XXS Saturday,
April 7, 1900.
[Subscription Terms,] pRICE TWOPENCE,

				

